# Oral health knowledge and oral hygiene practice among visually impaired subjects in Addis Ababa, Ethiopia

**DOI:** 10.1186/s12903-022-02199-x

**Published:** 2022-05-06

**Authors:** Wondwossen Fantaye, Abdela Nur, Getachew Kifle, Fasikawit Engida

**Affiliations:** 1 Community Dentistry Unit, Department of Dentistry, School of Medicine, College of Health Sciences, Addis Ababa University, Addis Ababa, Ethiopia; 2 Periodontology Unit, Department of Dentistry, School of Medicine, College of Health Sciences, Addis Ababa University, Addis Ababa, Ethiopia; 3 Orthodontics Unit, Department of Dentistry, School of Medicine, College of Health Sciences, Addis Ababa University, Addis Ababa, Ethiopia

**Keywords:** Oral health knowledge, Tooth brushing, Chewing stick, Visual impairment

## Abstract

**Background:**

Oral health is a global issue. It has an enormous impact on the overall health and well-being of an individual. In addition, many studies indicate visual impairment as one of the constraints for proper maintenance of oral hygiene. However, little is known about visually impaired individuals' oral health knowledge and behavior in Ethiopia, specifically in Addis Ababa. Therefore, this study is conducted to assess the oral health knowledge and related behaviors among participants with visual impairment in Addis Ababa, Ethiopia.

**Aim:**

This study aimed to assess the oral health knowledge and oral hygiene practice among visually impaired participants in Addis Ababa, Ethiopia. It is initially imperative to recognize the deficiency of data regarding the dental health care and needs of such visually impaired individuals in Ethiopia.

**Methods:**

It is a quantitative cross-section study design carried out at the Ethiopian National Association for the blind, located in Addis Ababa, the capital city of Ethiopia. A survey was conducted by convenience sampling of visually impaired library attendees' at the blind association. Sixty-five individuals, of which 46 males and 19 females, agreed to take part in the study. Of these, 30.8% had partial and 69.2% total visual impairment.

**Results:**

61.3% with totally and 72% with partially visually impaired scored high in the knowledge of caries causes respectively. The use of tooth brushing was confirmed by 42.2% with totally and 25% with partially visually impaired. The use of Traditional chewing sticks was confirmed by 57.7% with totally and 30% with partially visually impaired individuals. Moreover, both (Toothbrush and Traditional chewing stick) was used by 35.5% totally and 55% partially visually impaired individuals, respectively.

**Conclusions:**

The study showed the awareness about the causes of dental caries among the visually impaired was high. However, the majority of them had a significant misconception about the causes of dental caries.

**Supplementary Information:**

The online version contains supplementary material available at 10.1186/s12903-022-02199-x.

## Background

The World Health Organization (WHO) defines oral health as a branch of dental medicine that is concerned with being free of chronic mouth and facial pain, oral and throat cancer, oral sore, congenital disabilities such as cleft lip and palate, periodontal disease, tooth decay, and tooth loss, and other diseases and disorders that affect the mouth and oral cavity [1]. Oral health is essential because it is crucial for general health [2, 3]. Oral health, and in particular, oral pathology, could have important systemic repercussions. The state of constant inflammation, or even the bacterial blood circulation or bacterial products, could be a further cause of system pathologies [4]. In addition, oral diseases in terms of dental caries, periodontal diseases, and oral cancer are closely linked to personal behavior such as dietary habits, use of tobacco products, and oral hygiene practices. According to Kasi and Cobbs two broad classes of oral health-related behavior can be distinguished; oral health-enhancing (positive) behavior and oral health-compromising (risk) behavior. It also raises concern about how people practice oral health or their behaviors and maintains their oral health [5].

Oral disease is considered a public health problem since its prevalence is high. And its social impact is significant [6]. Oral diseases are among the most severe non-communicable chronic diseases that people suffer from and are the fourth most expensive disease to treat [7]. Furthermore, it is a global issue that affects human beings worldwide.

Blindness is defined by WHO as having a Visual acuity of less than 3/60 m or corresponding visual field loss in the better eye with the best possible correction; meaning that whilst a blind person could see 3 m, a non-visually impaired person could see 60 m. Visual impairment relates to a person's eyesight which cannot be corrected to normal vision [8].

According to the American Foundation for the Blind, the clinical parameters to define blindness from a legal perspective (legally blind) correspond to a central visual acuity of 20/200 or less in the better eye and/or a visual field of 20 degrees or less. Individuals diagnosed with legal blindness usually still possess some vision. Total blindness is defined as the inability to see anything at all with either eye [9]. The etiology of Visual Impairment varies with different population groups. According to the WHO, the most common cause of visual impairment in the developing world is untreated Cataracts (43%) and different Ocular diseases secondary to Diabetes Mellitus (24%) WHO, World Health Report [10]. Population-based surveys in Ethiopia reported cataracts as the main cause of blindness [11, 12]. The study from Butajira, Ethiopia, indicated that blindness is either preventable or curable in 74% of the cases [12].

The WHO has estimated that globally about 285 million people are visually impaired, and among these blind people are 39 million and 246 million with low vision [13]. While Bourne wrote 253 million people live with Vision Impairment: 36 million are blind, and 219 million have moderate to severe impairment [14]. Even though over 80% of global visual impairment is preventable or treatable, millions remain at risk of visual loss due to the lack of eye-care services [13].

Visual impairment has an impingement on oral health through physical, social, or informational barriers related to impairment, medical condition, and associated medical disorders. As a result, visually impaired people are challenged in their daily activities. The effects of blindness are many, but one of the most common is the inability of the individual to maintain Oral health [15].

Few studies have investigated oral health in visually impaired children, most of who live in Asia and Africa's poorest regions [16]. Poor oral hygiene, gingivitis, and periodontal diseases have been reported among visually impaired children in studies from India [16–18], Iran [19]. Turkey [20]. Sudan [21, 22]. Mann et al. suggested it can be due to their inability to visualize the plaque on tooth surfaces resulting in inadequate plaque removal and, therefore, the progression of dental caries and inflammatory disease of the periodontium [16, 23].

There is no information available regarding the oral health knowledge and oral hygiene practices with respect to visually impaired subjects in Addis Ababa, Ethiopia. The aim of the study is to assess the effect of visual impairment on the oral health knowledge and oral hygiene practices of individuals at the Ethiopian association for the blind. The findings are believed to provide valuable information that may be used as baseline for further investigation.

## Materials and methods

The study was carried out over two and half months during autumn 2018 at the Ethiopian National Association for the blind, located in Addis Ababa, the capital city of Ethiopia. Convenience sampling was used for the library attendees of the impaired individuals entering the study. Of these, 20 (30.8%) were with low vision, and 45 (69.2%) were totally visually impaired.

### Samples selection

Participants with total blindness, partial blindness, consent, library attendees at the association were included in the study. Whereas individuals with full vision sight and unwilling to participate were excluded.

### Survey procedures

By using interviewing questionnaires, the interviewers, 4 Dental Surgeons (1 Female, 3 Males) who are taking care of each category of the visually impaired individuals, were asked to collect data from the participants. These interviewers are Dental surgeons who are well trained on different general health issues. The participants were notified of the method and aim of the study and voluntarily participated. However, they were advised that they could withdraw from the interview at any time. The privacy and confidentiality assured ethical consideration of the discussion with each participant with documented informed consent. The questionnaire was read to participants, and participants' responses were in writing.

### Survey instrument

The study was conducted by distributing a pre-coded questionnaire constructed in English by the authors and then translated to local Amharic language to be clearly understood by the interviewers and the study participants. The questionnaire contained questions to assess socio-demographic characteristics, type of blindness. And, several variables related to knowledge of causes of dental caries, including tooth cleaning frequency, durations, methods of cleaning, and tooth cleaning tools shown in Table 1.

**Table 1:** Sample question for the assessment of oral hygiene practice

The following questions are related to oral hygiene methods, please listen to them carefully and reply only one response category, the one that suits you the best
How often do you brush your teeth?
Never
Once a week
1–2 times a week
Every other day
Once a day
2 times a day
3 times a day
5 times a day

### Measurements

Gender was assessed as male and female. The participants were also divided into totally or partially visually impaired; total visual impairment subjects cannot see anything, even light, and partially visually impaired issues have limited vision [24, 25]. The ages were grouped into ≤ 19 and ≥ 20 years old. The socioeconomic characteristics were divided into low and high education; low education subjects got their education at the primary school level, and high education subjects got their education at the secondary school and university level. Moreover, the participants were requested to evaluate their knowledge of the causes of dental caries. Caries can occur by improper cleaning; frequently consuming sweets; not visiting a dentist; having weak teeth; having worms in the tooth. A sum index of knowledge about caries was constructed (range 1–5) and reduced to a dummy variable, high expertise and low knowledge based on a Median split. Tooth cleaning tool was measured as toothbrush; traditional chewing stick; both and rinsing with water.

The frequency of tooth-cleaning using toothbrushes was measured as: less or equal to ≤ 2 times a day to more than two times a day; and more than three times a week. Brushing methods were assessed by asking "proper brushing, improper or just brushing. Type of brush was assessed as; Smooth medium, hard, and (I don't know)—duration of Brushing assessed as less than 3 min; more than 3 min.

Frequency of tooth-cleaning using Traditional stick was measured as: less or equal to ≤ 2 times a day to more than two times a day; and more than three times a week. Brushing methods were assessed by asking "proper brushing, improper or just brushing. Type of brush was assessed as; old, new, soft, and (whatever available). Duration of brushing assessed as two dummy variables were constructed yielding < 3 min and > 3 min.

In addition, Preference of Toothbrush and Traditional chewing stick was measured. The toothbrush was assessed as: It cleans appropriately; it is modern; it is comfortable. Traditional chewing stick was assessed as: It cleans properly; affordable, and it is religious and comfortable.

### Statistical analysis

The data were processed and analyzed using the Statistical Package for Social Sciences [26]. Frequency distributions of variables were computed separately for total and partially visually impaired individuals.

### Ethical considerations

The study proposal was submitted to the Department of Dentistry for ethical clearance, and written consent was obtained from the director of the blind association. In addition, a consent letter was carefully read to the participants before study commencement by the interviewers (4 Dental Surgeons).

In this concern, it has been stated to the participants that there is no direct benefit of their participation in the study. However, knowledge gained from the study may lead to the establishment of an oral health prevention program for visually impaired people (general population benefits) and about the confidentiality, that no information about the participants, or provided by them during the research will be disclosed to others without their written consent.

## Results

The total participants were 65 individuals, 46 males and 19 females, totally visually impaired were 45, and partially visually impaired were 20, respectively. Their ages ranged from 10 to 65, with a mean age of 27.2 years.

**Fig. 1:**
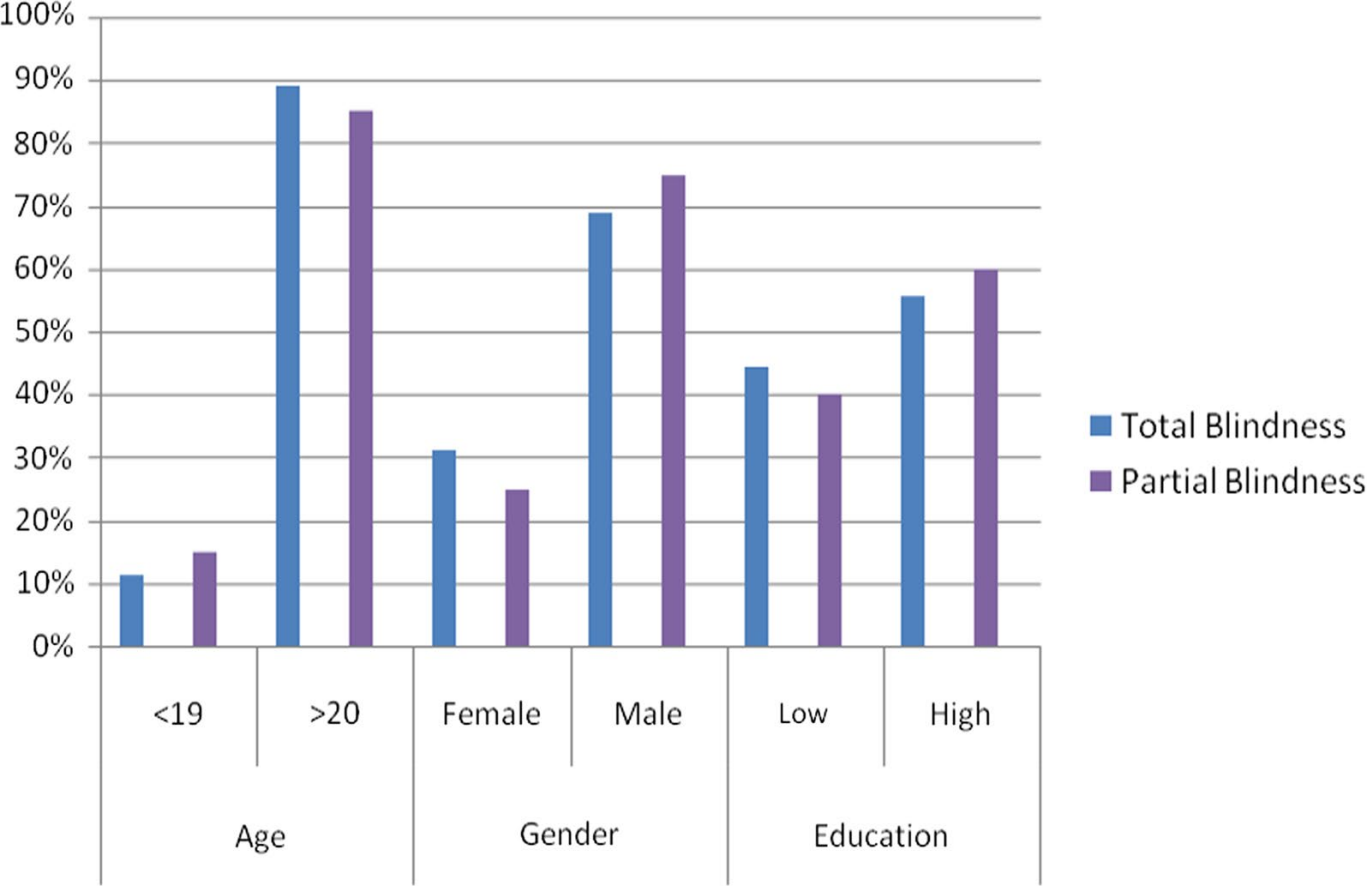
Percentage distribution (%) of the study participants, socio-demographic characteristics and types of blindness

Results regarding the percentage distribution and the number of study participants' socio-demographic characteristics and type of visual impairment are summarized in Fig. 1.

**Fig. 2:**
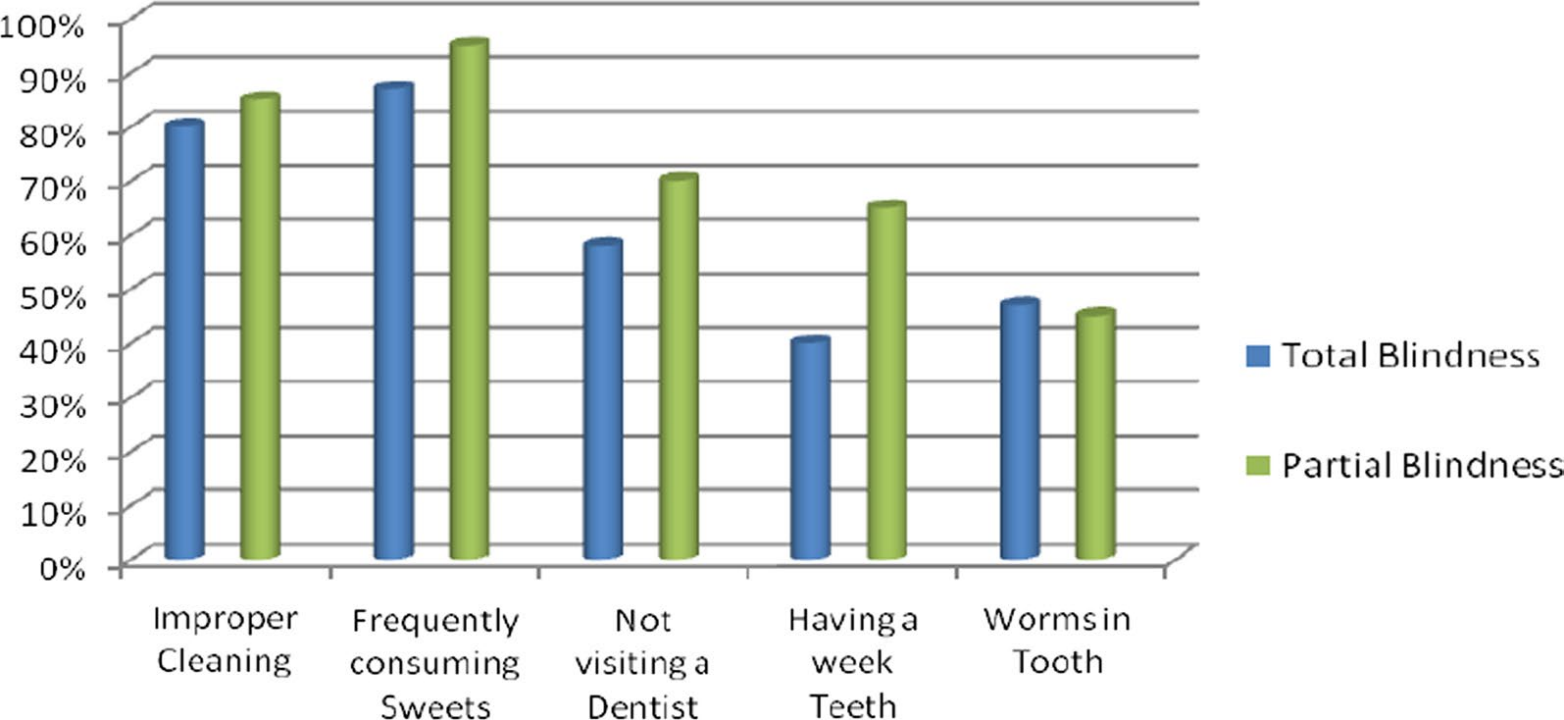
Percentage distribution (%) of the study participants, oral health knowledge items, causes of dental caries and types of blindness

The knowledge item was divided into causes of caries. A total of 45 (61.3%) totally visually impaired and 20 (72%) partially visually impaired scored high in caries' knowledge, respectively. The results regarding the percentage distribution of the study participants who confirmed specific causes of dental caries and type of visual impairment are shown in Fig. 2.

Tooth cleaning tool was measured as Toothbrush 19 (42.2%) totally and 5 (25%) partially visually impaired respectively. On the other hand, traditional chewing sticks were used 26 (57.7%) totally and 6 (30%) partially visually impaired. Moreover, both (toothbrush and traditional chewing sticks) were used by 16 (35.5%) totally and 11 (55%) partially visually impaired participants, respectively, and rinsing with water was 1 (2.2%).

**Fig. 3:**
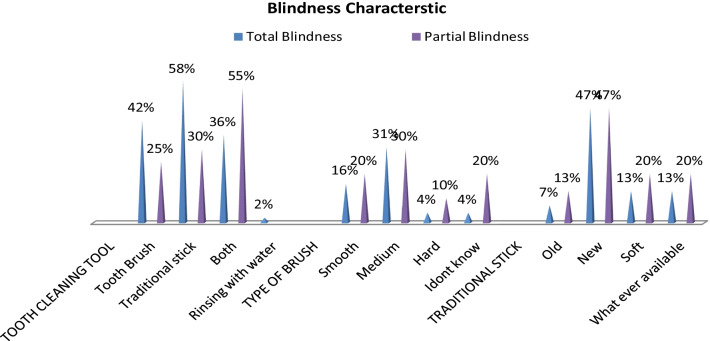
Percentage distribution (%) of the study participants, frequency of tooth cleaning tool, brush type and type of blindness

Percentage distribution (%) and numbers (n) of the study participants, according to frequency, tooth cleaning Tool, Brush type, and type of visual impairment, are summarized (Fig. 3).

Regular tooth brushing (less or equal to ≤ 2 times a day) was common in blind type groups, 20 (44.4%) totally and 5 (25%) partially visually impaired, respectively. Both types of blindness were equally responded16 (35.5%) totally and 6 (30%) partially visually impaired respectively used improper brushing methods. At the same time, only a few numbers showed proper brushing methods, 5 (11.1%) totally and 2 (10%) partially visually impaired, respectively. Duration of tooth brushing for < 3 min was responded among a totally 20 (44.4%) and 5(25%) partially visually impaired, respectively.

More than two-thirds of toothbrush user participants' primary reason for preferring toothbrush is that it is modern and has superior cleaning property. The numbers and percentages (%) distribution of the study participants according to frequency, a method of brushing and brush type, and type of visual impairment is as shown in Table 2.

**Table 2:** Percentage distribution (%) and numbers (n) of the study participants, according to brushing frequency, duration, preference of tooth brush, method of brushing and type of visual impairments

	Blindness characteristic			
	Total visual impairments		Partial visual impairments	
	[Participants n = 45]		[Participants n = 20]	
	%	n	%	n
*Brushing frequency*				
≤ 2 times a day	44.4	20	25	5
> 2 times a day	2.2	1	5	1
More than 3 times a week	8.8	4	20	4
*Brushing method*				
Proper brushing	11.1	5	10	2
Improper brushing	35.5	16	30	6
Just brushing	8.8	4	10	2
*Duration of brushing*				
< 3 min	44.4	20	45	9
> 3 min	11.1	5	5	1
*Preference of tooth brush*				
It cleans properly	13.3	6	15	3
It is modern	24.4	11	25	5
It is comfortable	17.7	8	10	2

Regular use of traditional chewing stick (less or equal to ≤ 2 times a day) was equally common in blind type groups, 20 (44.4%) totally and 9 (45%) partially visually impaired, respectively. Moreover, 12 (26.6%) totally and3 (15.0%) partially visually impaired respectively was responded by brushing with a traditional stick more than two times a day.

Approximately more than half the number of totally and 22 (55.5%) and 7 (35%) partially visually impaired who used traditional chewing sticks applied the proper brushing technique. In comparison, only a few numbers showed improper brushing 8 (17.7%) totally and 1 (5.0%) partially visually impaired. Whereas a few participants showed, just brushing 6 (13.3%) totally and 4 (8.8%) partially visually impaired, respectively.

A substantial proportion of the totally 24 (53.3%) and 7 (35%) partially visually impaired respectively reported use of Traditional chewing stick for more than 3 min. Brushing by traditional chewing stick less than 3 min was responded among totally 12 (26.6%) and 5 (25%) partially visually impaired respectively.

The majority, 32 (71%) totally and 10 (50%) partially visually impaired respectively, gave other reasons for reasonable prices, proper cleaning, comfort, and religion, for preferring traditional chewing stick.

The numbers and percentages (%) distribution of the study participants according to frequency, method of brushing, duration preference of using a traditional stick and type of visual impairment is as shown in Table 3.

**Table 3:** Percentage distribution (%) and numbers (n) of the study participants, according to frequency, duration, preference of traditional stick, technique of brushing and type of blindness

	Blindness characteristic			
	Total blindness [participants n = 45]		Partial blindness [participants n = 20]	
	%	n	%	n
*Frequency of brushing*				
Traditional chewing stick				
≤ 2 times a day	44.4	20	45	9
> 2 times a day	26.6	12	15	3
More than 3 times a week	8.8	4	–	–
*Brushing technique*				
Traditional chewing stick				
Proper brushing	55.5	22	35	7
Improper brushing	17.7	8	5	1
Just brushing	13.3	6	8.8	4
*Duration of brushing*				
Traditional chewing stick				
< 3 min	26.6	12	25	5
> 3 min	53.3	24	35	7
Preference of traditional chewing stick				
It cleans properly and affordable	71	32	50	10
It is religious	4.4	2	5	1
It is comfortable	4.4	2	5	1

## Discussion

The study was conducted to collect data on knowledge and behaviors of the visually impaired, investigate the findings and initiate preventive oral health education programs.

The study findings indicate a high proportion of both totally and partially visually impaired had correct knowledge, confirming that improper cleaning, frequently consuming sweets, and not visiting a dentist cause Dental caries. Similarly, a study conducted in Saudi Arabia indicated 71.5% total blind and 63.6% partial blinds scored high in the knowledge of caries [27].

A misconception among participants regarding the cause of dental caries signified a lack of knowledge about oral cavity and proper oral health.

It was found that worms in the tooth (46.7% totally and 45% partially visually impaired) respectively and having weak teeth (35.6% totally and 65% partially visually impaired) were considered causes of caries. Ahmad 2009 & Sabililillah 2016 also reported that dental and oral health knowledge in people with visual impairment is less due to lack of education in people with visual impairment, which affects their ability in gaining oral health knowledge [19, 28]. Scientific research shows that good oral health contributes to good general health; oral health care should therefore be part of health care [29].

The findings highlighted improper tooth brushing on the majority of the participants even though they were educated; completing at least secondary school. In addition, many visually impaired individuals found maintaining their oral hygiene more difficult due to their lack of vision which is a constraint to understanding and mastering techniques of oral hygiene practices [30]. Visual disorders affect oral health due to physical, social, or informational barriers related to impairment, medical condition, or a lack of customized information [31].

In Ethiopia, the traditional chewing stick, generally called the MEFAKIA, is used by most of the population. It was found that the Mefakia was found to be as effective as the toothbrush in oral cleaning [32]. A considerable number of participants from both totally and partially visually impaired frequently used traditional chewing sticks. This is consistent with a study from Sudan [33] and in line with Olsson's study in Ethiopia [32]. The traditional chewing stick can be recommended for use in Ethiopia since it is low-priced and customizable. Similar studies reported that wooden chewing sticks are extensively used among African populations for oral cleaning [34–38].

### Limitations of the study

Due to an insignificant number of female participants in the study, no comparison was made between males and females. However, the authors are aware that the sample population of this report may not be representative of all blind people in Addis Ababa, Ethiopia.

## Conclusion

The study demonstrated insufficient knowledge among the visually impaired about the cause of dental caries. Moreover, the findings highlighted improper tooth brushing among most participants even though they were educated completing at least secondary school. Traditional chewing stick was an important tooth brushing tool.

### Recommendation

The key to addressing issues with visual impairment is effective information delivery. Various modalities can be used to educate visually impaired individuals about oral hygiene and oral health. For instance, the audio method can be used to give education on oral health which is delivered only through sound and without face to face. By utilizing this method, proper oral hygiene methods and techniques can be recorded and broadcasted within the library compound on different local languages on a daily basis. The other way can be providing knowledge of dental health with the Braille method. Braille Dental Education (BDE) is a learning process in the oral health field devoted to the visually impaired or a person with a visual disability using Braille. The goal is to teach, train, develop, and accomplish appropriate knowledge and skills to visually impaired individuals to ensure self-reliance on maintaining their oral hygiene and oral health.

The Technology Related Assistance to Individuals with Disabilities Act of 1988 described an assistive technology device as "any item, piece of equipment, or product system, whether acquired commercially off the shelf, modified, or customized, that is used to increase, maintain, or improve functional capabilities of individuals with disabilities. “These Assistive technologies can be used also for visual impairment ranging from "high tech" to “low tech”. For examples; Kurzweil education.

The Kurzweil education offers various opportunities to deliver picture and text based oral hygiene instructions. This text-to-speech software can help those who are visually impaired to use computers and also can read scanned printed material. Kurzweil creates opportunities to convey picture and text based oral hygiene instructions.

A screen reader can also be used to target this demography; it is a software program which enables a visually impaired user to read the text that is displayed on the computer screen with a speech synthesizer or braille display. It can provide speech and Braille output for the most popular computer applications on a Personal Computer. It can help to navigate the Internet, write a document, read an email and create presentations from the office, remote desktop, or from home. Therefore, it can be used as platform and open access to customized oral hygiene maintenance instructions and oral health in general.

Since this is the first study of its kind in Ethiopia, the findings are believed to provide valuable information that may be used as a baseline for further studies. Moreover, this area of research deserves a more extensive investigation that should include oral examination and more individuals with of visual impairment.

## Supplementary Information

**Additional file 1.** Interview consent form.

## Data Availability

The datasets used and/or analyzed during the current study are available from corresponding author on reasonable request.

## Abbreviations

WHO: World health organization
BDE: Braille Dental Education
