# Supplementary material for: Oral health knowledge and oral hygiene practice among visually impaired subjects in Addis Ababa, Ethiopia
Source: BMC Oral Health. 2022 May 6;22:167. doi: 10.1186/s12903-022-02199-x (PMC9077845; doi:10.1186/s12903-022-02199-x)
Supplement: Supplementary file 1 — Additional file 1. Interview consent form. [file 12903_2022_2199_MOESM1_ESM.docx]

**Annex I**

**Interview Consent Form**

Research title: **Knowledge and oral health related behavior among visually impaired subjects in Addis Ababa, Ethiopia**

Research investigator: Dr. Wondwossen Fantaye

Research Participants number:

First of all, thank you for agreeing to be interviewed as part of the above research. Ethical procedures for this academic research were undertaken from the School of Medicine, Department of Dentistry institutional review board requires that interviewees explicitly agree to being interviewed and how the information contained in their interview will be used.

The interview will take **20 Minutes**. We don’t anticipate that there are any risks associated with your participation, but you have the right to stop the interview or withdraw from the research at any time.

This consent form is necessary for ensuring that you understand the purpose of your involvement and that you agree to the conditions of your participation.

Would you therefore **LISTEN** the accompanying information **NARRATED and** then provide verbal consent to certify that you approve the following?

• The responses will be recorded on the survey questionnaire
• Your response will be read back to you and given the opportunity to correct any factual errors
• Access to the interview responses will be limited to Dr.Wondwossen Fantaye ,academic colleagues and researchers with whom he might collaborate as part of the research process
• Any interview content, or direct response from the interview, that are made available through academic publication or other academic outlets will be anonymized so that you cannot be identified, and care will be taken to ensure that other information in the interview that could identify yourself is not revealed
• The actual completed survey questionnaire will be kept in a locked filling cabinet and the analyzed data will be stored in password protected storage device.

• Any variation of the conditions above will only occur with your further explicit
approval

All or part of the content of your interview may be used;
 In academic papers, policy papers or news articles
 On websites and in other media that we may produce such as spoken presentations
 On other feedback events
 In an archive of the research as noted above

**By approving to this form, you agree that;**

Voluntarily taking part in this research and understand that you don’t have to take part, and you can stop the interview at any time;

The interview response or extracts from it may be used as described above;

You have listened to the read information sheet;

You don’t expect to receive any benefit or payment for your participation;

You are able to ask any questions you might have, and understand that you are free to contact the researcher with any questions you may have in the future.

Contact information

Researcher/Data Collector signature: - Date: -

Name of researcher/Data Collector: -

*** The tittle of research is now revised to “**ORAL HEALTH KNOWLEDGE AND ORAL HYIGIENE PRACTICE AMONG VISUALLY IMPARED SUBJECTS IN ADDIS ABABA, ETHIOPIA.”**
